# AGNEP: An Agglomerative Nesting Clustering Algorithm for Phenotypic Dimension Reduction in Joint Analysis of Multiple Phenotypes

**DOI:** 10.3389/fgene.2021.648831

**Published:** 2021-04-26

**Authors:** Fengrong Liu, Ziyang Zhou, Mingzhi Cai, Yangjun Wen, Jin Zhang

**Affiliations:** ^1^College of Science, Nanjing Agricultural University, Nanjing, China; ^2^School of Data Science, University of Science and Technology of China, Hefei, China; ^3^Postdoctoral Research Station of Crop Science, Nanjing Agricultural University, Nanjing, China

**Keywords:** genome-wide association study, statistical power, clustering algorithms, principal component analysis, genetic structure

## Abstract

Genome-wide association study (GWAS) has identified thousands of genetic variants associated with complex traits and diseases. Compared with analyzing a single phenotype at a time, the joint analysis of multiple phenotypes can improve statistical power by taking into account the information from phenotypes. However, most established joint algorithms ignore the different level of correlations between multiple phenotypes; instead of that, they simultaneously analyze all phenotypes in a genetic model. Thus, they may fail to capture the genetic structure of phenotypes and consequently reduce the statistical power. In this study, we develop a novel method agglomerative nesting clustering algorithm for phenotypic dimension reduction analysis (AGNEP) to jointly analyze multiple phenotypes for GWAS. First, AGNEP uses an agglomerative nesting clustering algorithm to group correlated phenotypes and then applies principal component analysis (PCA) to generate representative phenotypes for each group. Finally, multivariate analysis is employed to test associations between genetic variants and the representative phenotypes rather than all phenotypes. We perform three simulation experiments with various genetic structures and a real dataset analysis for 19 *Arabidopsis* phenotypes. Compared to established methods, AGNEP is more powerful in terms of statistical power, computing time, and the number of quantitative trait nucleotides (QTNs). The analysis of the *Arabidopsis* real dataset further illustrates the efficiency of AGNEP for detecting QTNs, which are confirmed by The *Arabidopsis* Information Resource gene bank.

## Introduction

Genome-wide association study (GWAS) is a powerful tool for exploring associations between genetic variants and phenotypes. To date, GWAS has been successfully applied to human, plant and animal genetic research, to identify thousands of genetic variants related to phenotypes or diseases. Common statistical methods only test the relationships between a single phenotype and loci, that is, only one phenotype is analyzed at a time. Compared to univariate analysis, joint analysis of multiple phenotypes can improve the accuracy and efficiency of the test by using more information from multiple phenotypes (Allison et al., 1998; Zhou and Stephens, 2014), which can be very advantageous for two reasons (Allison et al., 1998; Zhou and Stephens, 2014). First, it promotes computing efficiency. Most of the multi-phenotype methods perform the test for association with all traits, instead of analyzing phenotypes one by one. Joint analysis greatly reduces calculating time and promotes analytical efficiency. Second, multivariate analysis increases statistical power by using genetic structure and potential information among different traits rather than ignoring them as in univariate analysis (Ferreira and Purcell, 2009; Huang et al., 2011). Currently, more and more multivariate analyses have been put forward to analyze the related phenotypes.

The previous studies illustrated that more than 4.6% of single nucleotide polymorphism (SNPs) and 16.9% of genes are reported to be significantly associated with more than one trait (Solovieff et al., 2013). Due to the fact that the joint analysis of multiple phenotypes is more consistent with biological theory (van der Sluis et al., 2013), many multivariate methods have been proposed (Galesloot et al., 2014). O’Brien’s method (O’Brien, 1984), one of the earliest methods of jointly analyzing multiple phenotypes, can be used to integrate the results of univariate association tests. If the means of individual statistics are homogeneous, O’Brien’s method is more effective among linear combination statistics. Multivariate analysis of variance (MANOVA) (Cole et al., 1994) is a classic method of analyzing multiple phenotypes that jointly tests whether the independent variables explain the variance of the dependent variables statistically significant at the same time. Subsequently, Multiphen (O’Reilly et al., 2012) and TATES (van der Sluis et al., 2013) are powerful to test associations between genetic variants and corresponding multiple traits. Under the framework of linear mixed models, multi-trait mixed model (Korte et al., 2012) and multivariate linear mixed model (Zhou and Stephens, 2014) are proposed, which take into account the variance components of multiple phenotypes and the population structure in GWAS.

However, established procedures for analyzing multiple phenotypes face several challenges from the following perspectives. First, computing is infeasible. Hundreds and thousands of phenotypes are being collected in biological experiments and surveys. However, most methods become computationally intractable or hard to implement as the number of phenotypes increases (Dahl et al., 2016). Second, estimates are inaccurate. The complexity and the number of parameters increase sharply in joint analysis of more than 10 phenotypes, and hence accuracy and statistical stability decrease (Solovieff et al., 2013). Finally, most multivariate algorithms simultaneously analyze all phenotypic data and thus might ignore different level of correlation or homogeneous genetic basis among traits, resulting in an unsatisfactory power (Liang et al., 2018).

Clustering algorithm is an alternative method of overcoming these challenges. It aims to maximize homogeneity within a cluster so that similarity is greater between elements in the same cluster than those in different clusters. As the dimension of the data is reduced by clustering, temporal and spatial complexity decreases. In addition, the intragroup phenotypic correlation is stronger than the intergroup correlation, which improves the efficiency and accuracy of the statistical test. Therefore, clustering is great importance to the study of the joint analysis of high-dimensional phenotypes. Recently, Sha et al. (2019) proposed the cluster linear combination (CLC) method, which groups phenotypes and then analyzes quadratic combination of individual data. CLC takes full advantage of similar genetic information in the same group. However, CLC does not work well with negative or mixed correlations.

In this study, we propose a new method agglomerative nesting clustering algorithm for phenotypic dimension reduction analysis (AGNEP), which uses an agglomerative nesting (AGNES) clustering algorithm to group multiple correlated phenotypes and then applies principal component analysis (PCA) to generate representative phenotypes for each group. Finally, MANOVA is employed to test associations between genetic variants and the representative phenotypes rather than all phenotypes. In three simulation experiments, we consider six scenarios under three kinds of genetic structures to compare the performance of different methods: MANOVA, analysis of variance (ANOVA), a hierarchical clustering method with mean representative phenotypes (HCMM), AGNEP, AGNES with mean representative phenotypes (AGNEm), and AGNES with median representative phenotypes (AGNEmed). All of these methods are applied to analyze 19 traits of *Arabidopsis* real dataset. AGNEP is validated by the analysis of real dataset and the series of simulation experiments.

## Materials and Methods

### Genetic Model

Consider the multivariate linear model:

(1) $$Y_{\left(d\times n\right)}=\alpha W_{\left(d\times n\right)}+B_{\left(d\times 1\right)}X_{\left(1\times n\right)}+E_{\left(d\times n\right)}$$

where *Y*_*d*×*n*_ = (*Y*_1_,…,*Y*_*d*_)^*T*^ is a *d* × *n* matrix of phenotypes, *n* is the number of individuals and *d* is the number of phenotypes; *Y*_*i*_ = (*y*_*i*1_,…,*y*_*in*_)^*T*^ is the *i*^*th*^ phenotype of *n* individuals. α is the intercept and *W*_*d × n*_ is a *d* × *n* matrix with elements of 1. *B* is a *d*-vector of effect sizes for the *d* phenotypes, which are considered as fixed effects. *X*_1×*n*_ = (*x*_1_,…,*x*_*n*_) is an *n*-vector of genotypes for a particular marker, and *x_j_* is denoted as the number of minor alleles that the *j*^*th*^ individual carries at the variant. *E*_(*d*×*n*)_∼*MN*_(*d*×*n*)_(0,*V*,*I*_*n*_)is a *d* × *n* matrix of residual error. *MN*_*d*×*n*_(0,*V*,*I*_*n*_) denotes the *d* × *n* matrix normal distribution with mean 0, row covariance matrix *V* (a *d* × *d* symmetric matrix of environmental variance component) and column covariance matrix *I_n_* (an *n* × *n* identity matrix).

### Clustering Algorithms

Generally, hundreds or even thousands of phenotypes are cataloged from biological experiments and surveys. However, either these phenotypic data are analyzed separately by univariate analysis, or all phenotypes are analyzed without distinction. This creates some challenges for the statistical analysis, such as a reduction in statistical power, inflexibility in the computational analysis, a high computing time, and so on. From the perspective of multi-phenotype joint analysis, grouping high-dimensional phenotypic data by clustering algorithms is an alternative to overcome above challenges (Fung, 2001). Here we integrate clustering algorithms, AGNES into analysis of multiple phenotypes.

Hierarchical clustering algorithm creates a tree-like cluster structure based on the similarity between samples. In general, two partitioning strategies are possible according to the direction of hierarchical decomposition, that is, agglomerative (bottom up) and divisive (top down). The agglomerative method starts with all samples in their own clusters and then groups two clusters with the greatest similarity until only one cluster remains. The divisive method adopts an inverse procedure with agglomerative method (Liang et al., 2018).

AGNES is a typical hierarchical clustering algorithm, which implements bottom-up strategy until a preset criterion is satisfied (Deng et al., 2018). The similarity between *Y_i_* and *Y_j_* is evaluated by formula (2). The minimum distance is calculated by formula (3) to measure the similarity of clusters *c_i_* and *c_j_* (Murtagh and Legendre, 2014).

(2) $$dist\left(Y_{i},Y_{j}\right)={||Y_{i}-Y_{j}||}_{2}=\sqrt{\sum_{t=1}^{n}{\left|y_{\left(it\right)}-y_{\left(jt\right)}\right|}^{2}}$$

(3) $$dist_{min}\left(c_{i},c_{j}\right)=\underset{p\in c_{i},q\in c_{j}}{min}dist\left(p,q\right)$$

where *Y_i_* is the *i*^*th*^ phenotype; *c*_*i*_ = (*c*_*i*1_,…,*c*_*in*_)^*T*^ is the *i*^*th*^ cluster; *p* is a sample belonging to cluster *c_i_*, and *q* is a sample belonging to cluster *c_j_*.

### The Optimal Number of Clusters *K*

In this study, the optimal number of clusters *K* is calculated according to the maximum silhouette coefficient *s*, which is an index used to evaluate the clustering algorithm (Rousseeuw, 1987). The silhouette coefficient combines two factors, cohesion and resolution. Assuming all phenotypes are divided into *K* clusters by using AGNES, for each sample, we assume that *Y_i_* belongs to the cluster *c_k_*, we can calculate the silhouette coefficient *s* as formula (4):

(4) $$s\left(i\right)=\frac{b\left(i\right)-a\left(i\right)}{max\left(b\left(i\right),a\left(i\right)\right)}$$

$$a(i)=\{\begin{array}{cc} \frac{1}{|c_{k}|-1}\sum_{p\in c_{k},p\neq Y_{i}}dist(Y_{i},p), & |c_{k}|>1\\ 0, & |c_{k}|=1 \end{array}$$

$$b\left(i\right)=\underset{c_{d}\neq c_{k}}{min}dist\left(Y_{i},c_{d}\right)=\frac{1}{\left|c_{d}\right|}\sum_{q\in c_{d}},\left(Y_{i},q\right)$$

where *s*(*i*) is the silhouette coefficient of the sample *Y_i_*, *s*(*i*) ranges from −1 to 1, and |*c*_*k*_| is the number of phenotypes in cluster *c_k_*.

Obviously, *s*(*i*) close to 1 indicates that the distance within a cluster is small and the distance between clusters is large, that is, relatively better clustering results. The silhouette coefficient*s* is the average of silhouette coefficient of all samples, $s=d^{-1}\sum_{i=1}^{d}s\left(i\right)$. The optimal classification, say *K* clusters, is determined according to the maximum characteristics of the silhouette coefficient. In this study, the number of clusters *K* ranges from 2 to *d*−1, which means two situations are not considered, each phenotype is a cluster, and all phenotypes are clustered into one cluster.

### Representative Phenotypes of Clusters

In the following multivariate analysis, representative phenotype(s) are analyzed instead of all phenotypes by three ways: (i) the mean of each group (AGNEm), (ii) the median of each group (AGNEmed), and (iii) the top principal components of each group (AGNEP).

We scale each phenotype for each cluster and define the representative phenotype for the *k*^*th*^ cluster as the average or median phenotypic value within the group using formula (5) and (6):

(5) $$Y_{mean}^{k}=\frac{1}{\left|c_{k}\right|}\sum_{Y_{i}\in c_{k}}Y_{i}$$

(6) $$Y_{median}^{k}=\underset{Y_{i}\in c_{k}}{median}Y_{i}$$

In addition, top *m* principal components $Y_{PCA}^{k}=\left(Y_{PCA}^{k1},\ldots ,Y_{PCA}^{km}\right)$ with a cumulative contribution rate over 85% (Xue, 2007) are regarded as the representative phenotypes for the *k*^*th*^ cluster.

### Experimental Materials

Three simulation experiments are conducted to evaluate the performances of AGNEP and other methods. We generate genotypes according to the minor allele frequency in the interval [0.1, 0.5] under Hardy–Weinberg equilibrium. The simulation datasets contain *n* = 5000 individuals with *m* = 10,000 genetic variants, which are generated by using the factor model (Sha et al., 2019). We consider two scenarios for each simulation, including 10 quantitative trait nucleotides (QTNs) for scenario 1 and 50 QTNs for scenario 2.

**FIGURE 1:**
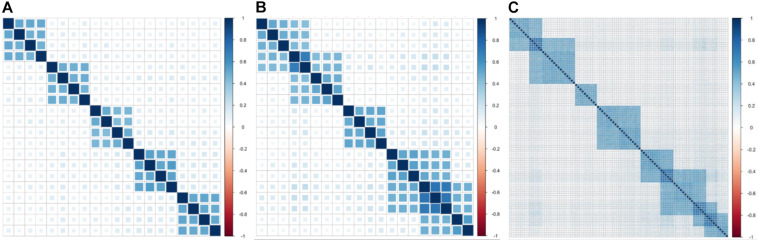
Genetic correlations for three simulation experiments. **(A)** Five independent clusters (simulation I). **(B)** Five dependent clusters (simulation II). **(C)** Eight dependent clusters of high-dimensional phenotypes (simulation III).

In simulation experiment I, 20 phenotypes are divided into five independent clusters (Table 1). Each cluster consists of four phenotypes based on genetic correlation (Figure 1A). In simulation experiment II, we consider a pervasive genetic structure. The adjacent clusters have overlapping phenotypes, and the overlapped phenotypes share the same or similar genetic basis. Twenty phenotypes are divided into five correlated clusters (Table 1). Group 1 and group 2 share two phenotypes, group 3 is independent with the other groups, and group 4 shares three phenotypes with group 5 (Figure 1B). In simulation experiment III, we focus on high-dimensional phenotypes with more complex correlations. All 100 phenotypes are divided into eight phenotypic groups. The genetic correlations are exhibited in Figure 1C. The high-dimensional correlations are more complicated than the correlations in the previous two simulation experiments.

**TABLE 1:** Different genetic structures for three simulation experiments, including five independent clusters (simulation I), five dependent clusters (simulation II), and eight dependent clusters of high-dimensional phenotypes (simulation III).

Simulation experiments	Simulation setting								
	Clustering	1	2	3	4	5	6	7	8
I	No. of phenotypes	1–4	5–8	9–12	13–16	17–20			
II	No. of phenotypes	1–5	4–8	9–12	13–18	16–20			
III	No. of phenotypes	1–15	10–30	31–40	41–60	61–75	70–90	85–95	90–100

#### *Arabidopsis* Real Dataset

**FIGURE 2:**
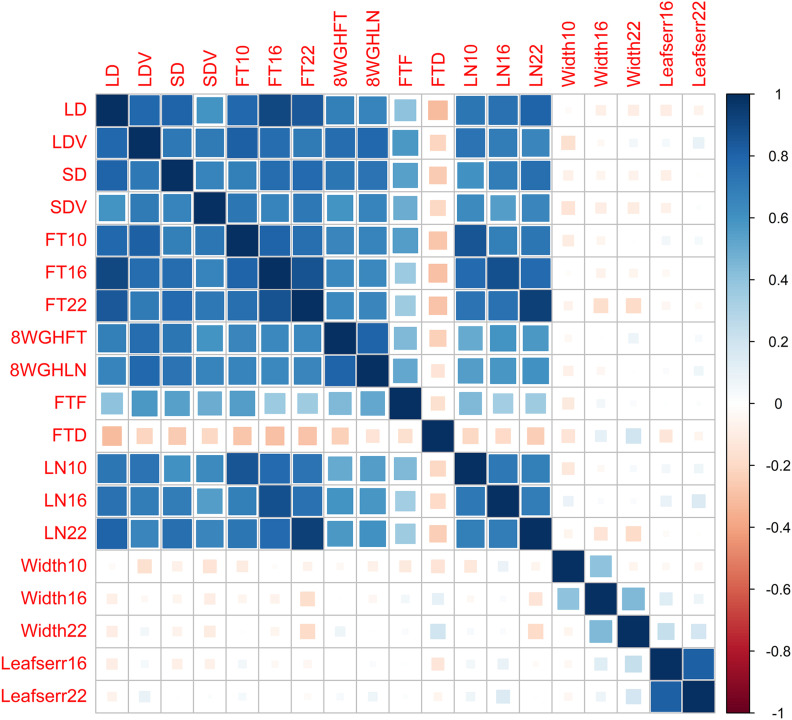
Genetic correlations between 19 phenotypes in the *Arabidopsis* dataset.

We reanalyze the *Arabidopsis thaliana* (Atwell et al., 2010) dataset, including 199 diverse inbred lines, each of which has 216,130 SNPs and 107 phenotypes. To evaluate the performance of different methods, we focus on 19 quantitative phenotypes: days to flowering under long days (LD), days to flowering under LD with vernalization (LDV), days to flowering under short days (SD), days to flowering under SD with vernalization (SDV), days to flowering at 10, 16, and 22°C (FT10, FT16, and FT22), days to flowering with 8 weeks vernalization in greenhouse (8WGHFT), leaf number at flowering with 8 weeks vernalization in greenhouse (8WGHLN), days to flowering in field (FTF), diameter of plants at flowering in field (FTD), leaf number at 10, 16, and 22°C (LN10, LN16, and LN22), plant diameter at 10, 16, and 22°C (Width10, Width16, and Width22), and presence of leaf serration at 16 and 22°C (Leafserr16 and Leafserr22). We filter out SNPs with minor allele frequency less than 5% and each individual with missing phenotypic data. After quality control, the data consist of 206,603 SNPs and 137 individuals. The genetic structure of the phenotypic data is shown in Figure 2.

## Results

### Simulation Results

To evaluate the performance of the following multivariate methods (MANOVA, HCMM, AGNEP, AGNEm, and AGNEmed) and univariate method (ANOVA), we conduct three simulations: independent phenotypic groups in simulation I (Figure 1A), correlated groups in simulation II (Figure 1B), and high-dimensional phenotypes divided into eight groups in simulation III (Figure 1C).

#### Statistical Power for Detection

**FIGURE 3:**
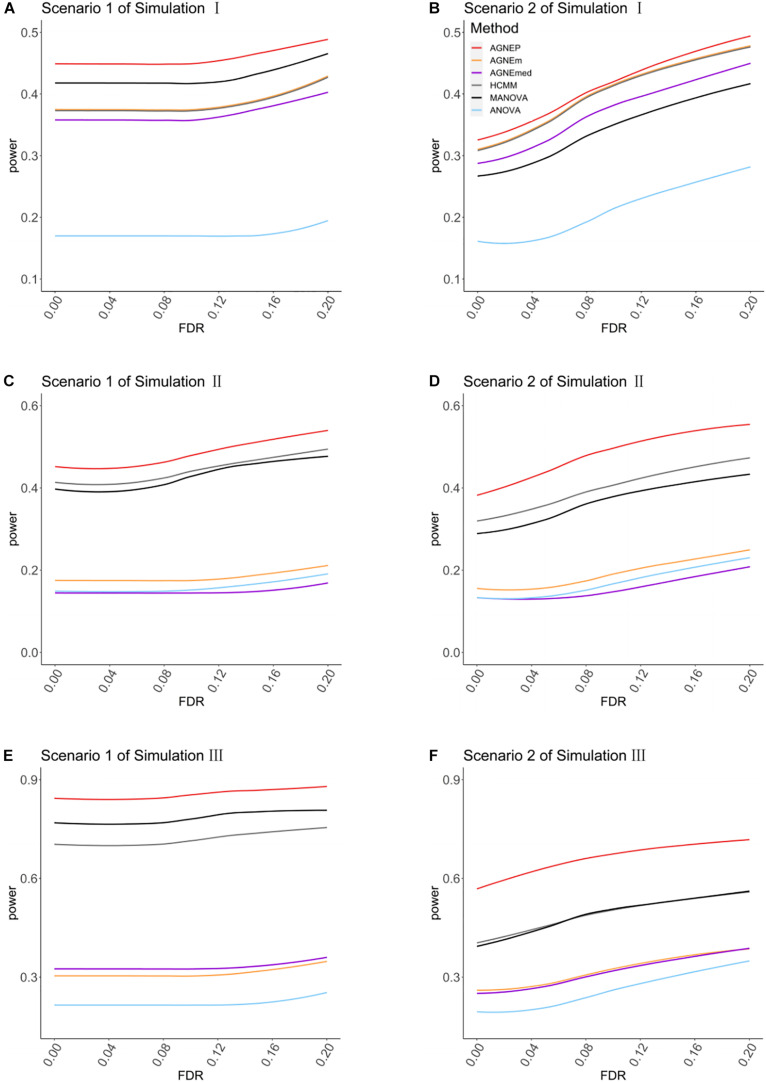
The comparison of the power for AGNEP and established approaches. **(A,B)** The powers of simulation experiment I are presented. **(C,D)** The powers of simulation experiment II are presented. **(E,F)** The powers of simulation experiment III are presented. Scenario 1 and 2 indicate that 10 and 50 QTNs are simulated in the three simulations, respectively.

In the three simulations, 10 (scenario 1) and 50 (scenario 2) QTNs are simulated in each dataset. For simulation I (independent groups), Figures 3A,B show the significant advantages of all multivariate analysis over the univariate analysis (ANOVA). According to the optimal silhouette coefficient of clustering algorithm (Supplementary Figure 1), the power under various FDR is higher for AGNEP than the other methods in simulation I. MANOVA easily captures the independent genetic structure of 10 QTNs (Figure 3A) and has slightly higher power than HCMM, AGNEm, and AGNEmed. In scenario 2, the multivariate analysis based on clustering algorithm obviously outperforms than MANOVA (Figure 3B). The clustering results for AGNEm and HCMM are completely consistent with the optimal silhouette coefficient, thus, these two methods have the same power, and their curves are overlapping in Figures 3A,B. From the results of simulation I, we conclude that AGNEP seems slightly more robust and multivariate algorithms easily capture genetic information for independent groups.

For simulations II (related groups) and III (high-dimensional related groups), the powers of almost all multivariate algorithms are significantly higher than that of the univariate analysis (ANOVA; Figures 3C–F). AGNEP has higher power and more significant detection in simulations II and III, which is followed by HCMM, MANOVA, AGNEm, AGNEmed, and ANOVA. In addition, the results of simulations II and III show that the power of AGNEm and AGNEmed are even worse than MANOVA and similar to ANOVA. It is evident that different representative phenotypes achieve significantly different results under the same clustering algorithm, and PCA appears to be a powerful tool for flexibly taking full advantage of potential information. Moreover, this difference becomes more and more obvious with the increase in the number of phenotypes, the complexity of the genetic structure, and the number of QTNs. The results of the three simulations demonstrate the superior power of AGNEP over all the other methods under various genetic structures.

#### Computing Time

**FIGURE 4:**
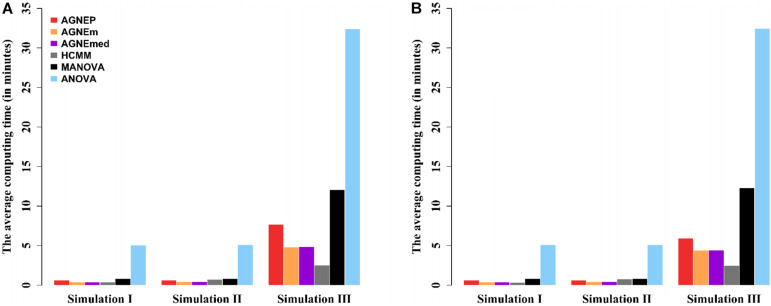
The average computing time (in minutes) of the six methods in three simulations. Scenario 1 **(A)** and 2 **(B)** indicate that 10 and 50 QTNs are simulated, respectively.

The computing times of the different methods in the three simulations are shown in Figure 4. For analyses of multiple phenotypes based on different clustering algorithms, the computing times are in the same magnitude, which are less than MANOVA and ANOVA. However, as the number of phenotypes increases, the differences among the methods are more and more obvious. The results of the three simulations illustrate that AGNEP effectively captures potential information and reduces the computing complexity. In particular, AGNEP is recommended for high-dimensional phenotypes and complex related structures.

### Real Data Analysis

To further evaluate the performance of the different methods, we analyze an *Arabidopsis* real dataset with 19 quantitative phenotypes including LD, LDV, SD, SDV, FT10, FT16, FT22, 8WGHFT, 8WGHLN, FTF, FTD, LN10, LN16, LN22, Width10, Width16, Width22, Leafserr16, and Leafserr22. All phenotypes are related to flower, leaf, plant growth, and the presence of leaf serration. After filtering, the dataset consists of 137 samples and a total of 206,603 SNPs. The genetic correction of the phenotypic data is shown in Figure 2.

#### QTNs Detected

**FIGURE 5:**
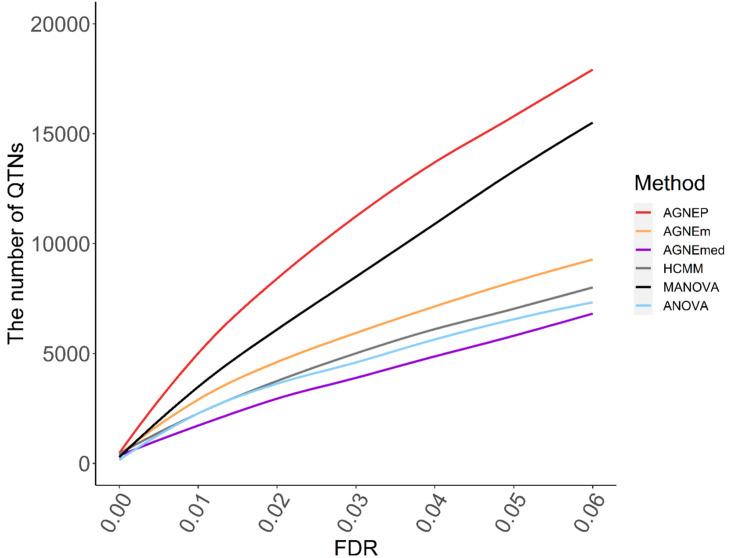
Numbers of QTNs under various FDR detected by six methods for the *Arabidopsis* dataset.

The numbers of putative QTNs for the six different methods are calculated by 10 permutations (Figure 5). Based on the maximum silhouette coefficient, AGNEP detects more putative QTNs than the other five methods, and the other multivariate algorithms and ANOVA have relatively poor detection ability. The results of the *Arabidopsis* real dataset show similar trends to simulation III. This may result from that the genetic structures are relatively complex, and the other methods cannot effectively capture this type of information, so their performances are not satisfactory.

#### Manhattan Plots

Manhattan plots of the *Arabidopsis* analysis are shown in Supplementary Figures 2,3. For ANOVA (Supplementary Figure 2), the QTNs related to phenotypes associated with flower and plant growth can be detected, whereas the QTNs related to other phenotypes have relatively low *P*-value. The results of statistical tests of AGNEP, AGNEm, AGNEmed, and HCMM (Supplementary Figure 3) show similar patterns, and several genomic regions reach the Bonferroni corrected threshold (−log_10_(0.001/206603) = 8.3151). According to the results for confirmed *Arabidopsis* genes, MANOVA detects more false associated SNPs. Therefore, compared to the univariate method, multivariate methods have the ability to increase statistical power. Moreover, multivariate methods based on the clustering algorithm further improve detection ability and accuracy by using information about complex genetic structure.

#### Genomic Patterns

**FIGURE 6:**
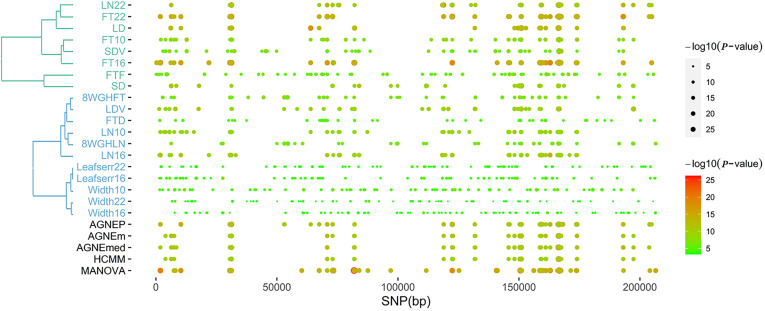
The plot of top 100 significant SNPs of six methods in the analysis of 19 traits. (Left) The hierarchical cluster diagram of 19 traits. (Right) The bubble chart of *P*-value.

According to the results of the 19 traits of *Arabidopsis*, all significant QTNs are listed in Figure 6 as hot spots, which illustrate information about the overall genomic patterns of significant SNPs (QTNs) on multiple traits. Almost all multivariate methods have the similar pattern. Compared to univariate method, multivariate methods easily identify associations between QTNs and phenotypes. This figure shows the genetic basis of functional relationships between phenotypes. These hot spots would be the primary targets for functional analysis and for genetic improvement by selection.

#### Confirmed Genes

**FIGURE 7:**
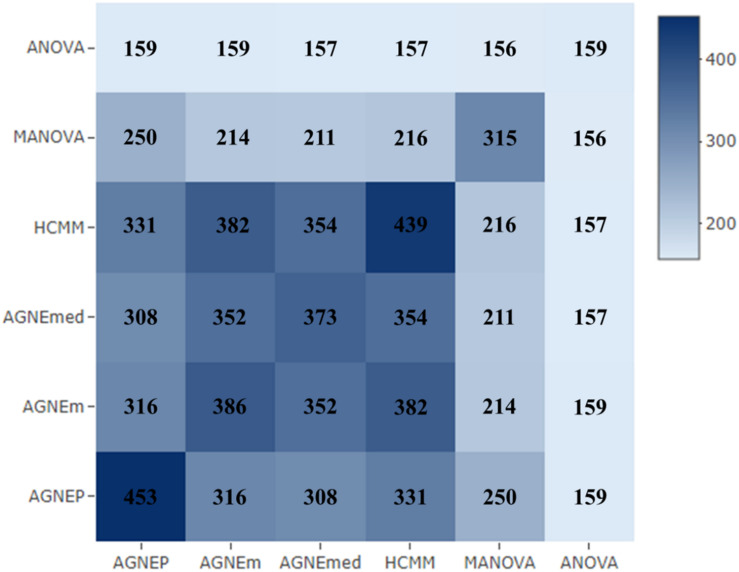
The heat map of confirmed genes for the six methods in analysis of the *Arabidopsis* real dataset. The darker the square, the greater the number of confirmed genes detected two methods.

To further validate the AGNEP method, we compare the number of candidate genes detected by six methods for the *Arabidopsis* dataset. All SNPs under 0 FDR within 20 kB of each putative QTN are used to mine the candidate genes by The *Arabidopsis* Information Resource^1^. Table 2 shows the quantity of confirmed genes for all approaches (Hagemann and Gleissberg, 1996; Wang et al., 2003; Nikovics et al., 2006; Albayrak et al., 2012; Nakayama et al., 2012). AGNEP detects the largest number of confirmed genes, 453, followed by HCMM (439), AGNEm (386), AGNEmed (373), MANOVA (315), and ANOVA (159). A heat map (Figure 7) illustrates the confirmed candidate genes simultaneously detected by two methods. It is obvious that the multivariate methods detect more identical confirmed genes than the univariate method (ANOVA). Furthermore, multivariate methods based on a clustering algorithm, say AGNEP, AGNEm, AGNEmed, and HCMM, detect more than 350 confirmed genes.

**TABLE 2:** Average computing time (in minutes) and number of confirmed genes in analysis of the *Arabidopsis* dataset by six different methods.

Method	Number of confirmed genes	Computing time
AGNEP	453	91.33
AGNEm	386	113.49
AGNEmed	373	95.64
HCMM	321	105.05
MANOVA	315	110.72
ANOVA	159	788.15

#### Computing Time

The computing time of each approach for the 19 *Arabidopsis* traits is listed in Table 2. Apparently, all the multivariate methods are faster than the univariate method, which consumes about seven to eight times longer than the multivariate methods. The multivariate analysis greatly reduce the calculating time and promotes analytical efficiency. AGNEP and AGNEmed have the shortest running time, less than 100 minutes; HCMM, AGNEm, and MANOVA have moderate computing times. All in all, AGNEP not only performs best in QTNs detection, but also has the fastest computing speed, which is validated by the analysis of the real dataset.

## Discussion

In this study, we propose a new method called AGNEP, which applies AGNES clustering algorithms and PCA to detect genetic associations between SNPs and multiple phenotypes in GWAS. The results of three simulations and a real data analysis indicate the merits of AGNEP. There are three main advantages. First, AGNEP easily captures the correlation of multiple phenotypes by clustering methods, which increases statistical power in analysis of simulations and *Arabidopsis* dataset (Figures 3, 5). Second, the detection accuracy of AGNEP is significantly improved. From the *Arabidopsis* dataset, AGNEP detects the most confirmed genes, obviously more than the other established methods. Third, because of the decrease in phenotypic dimension and the optimization of representative phenotypes, AGNEP enjoys fast computing speed, even with high-dimensional phenotypes and complex genetic structures.

To further validate the new method, we incorporate representative phenotypes into seven different clustering methods, including K-means, PAM, CLARA, HCDS, HCM, FCM, and EM algorithms. All of these methods are used to reanalyze the simulated datasets and *Arabidopsis* real data. The PCA-based methods are more robust than the methods, MANOVA and ANOVA from the perspective of power (simulation results, Supplementary Figure 4; *Arabidopsis* results, Supplementary Figure 5), efficiency (Supplementary Table 1), and detection of confirmed genes (Supplementary Table 2). However, all of these methods perform slightly worse than AGNEP in the simulations and real data analysis. Furthermore, CLC is used to comparing, which appears a tremendous increase in computational burden along with permutation and the number of phenotypes, and thus the simulation I and II datasets are analyzed. Nevertheless, the performance of CLC is unsatisfactory in terms of statistical power and efficiency.

Essentially, the representative phenotypes of PCA are linear combinations of individual phenotypic data in the same cluster. When the cluster consists of highly positively correlated phenotypes, all the linear combinations can represent the cluster reasonably well (Bühlmann et al., 2013; Shah and Samworth, 2013). To further validate PCA combinations, the mixed (both positive and negative) correlations are induced to simulation II. The PCA-based methods are better than the mean and median, and ANOVA has the lowest power (Supplementary Figure 7). For mixed and complex correlated phenotypes, the results demonstrate the good performance of the PCA combinations as well (Figure 3 and Supplementary Figure 7). This is because the PCA combinations consist of the most within-cluster information and reduce the phenotypic dimensions. It is necessary to further explore other representative phenotypes forms, such as quadratic and non-linear combinations.

With the development of life sciences and biotechnology, genetic data is becoming larger in scale and more complicated. How to cluster phenotypes efficiently and accurately is very important. In this study, the silhouette coefficient is a key index for evaluating the clustering model and determining the optimal number of clusters. In addition to the silhouette coefficient, many other criteria can be used to evaluate the model, such as Calinski-Harabaz, Dunn validity, and Davies-Bouldin. Silhouette coefficient is recommended according to empirical analysis.

## Data Availability Statement

The Arabidopsis data used for the analysis described in this manuscript was obtained from http://www.arabidopsis.usc.edu/.

## Author Contributions

JZ conceived and supervised the study and wrote and revised the manuscript. FL and ZZ performed all experiments, analyzed the data, and wrote the manuscript. MC and YW mined candidate genes from The *Arabidopsis* Information Resource in the *Arabidopsis* data analysis and created all figures and tables. All authors reviewed the manuscript.

## Conflict of Interest

The authors declare that the research was conducted in the absence of any commercial or financial relationships that could be construed as a potential conflict of interest.

## Footnotes

1 https://www.arabidopsis.org/

## Abbreviations

GWAS: genome-wide association study
SNP: single nucleotide polymorphism
QTN: quantitative trait nucleotide
PCA: principal component analysis
AGNES: agglomerative nesting clustering algorithm
AGNEm: AGNES with mean representative phenotypes
AGNEmed: AGNES with median representative phenotypes
AGNEP: AGNES for phenotypic dimension reduction analysis
ANOVA: analysis of variance
MANOVA: multivariate analysis of variance
CLC: cluster linear combination
HCMM: a hierarchical clustering method with mean representative phenotypes.
