# Supplementary material for: AGNEP: An Agglomerative Nesting Clustering Algorithm for Phenotypic Dimension Reduction in Joint Analysis of Multiple Phenotypes
Source: Front Genet. 2021 Apr 26;12:648831. doi: 10.3389/fgene.2021.648831 (PMC8107386; doi:10.3389/fgene.2021.648831)
Supplement: Supplementary file 1 [file Presentation_1.pdf]

# Supplementary

## AGENP: Agglomerative nesting clustering algorithm for phenotypic dimension reduction in joint analysis of multiple phenotypes

# Supplementary Figures

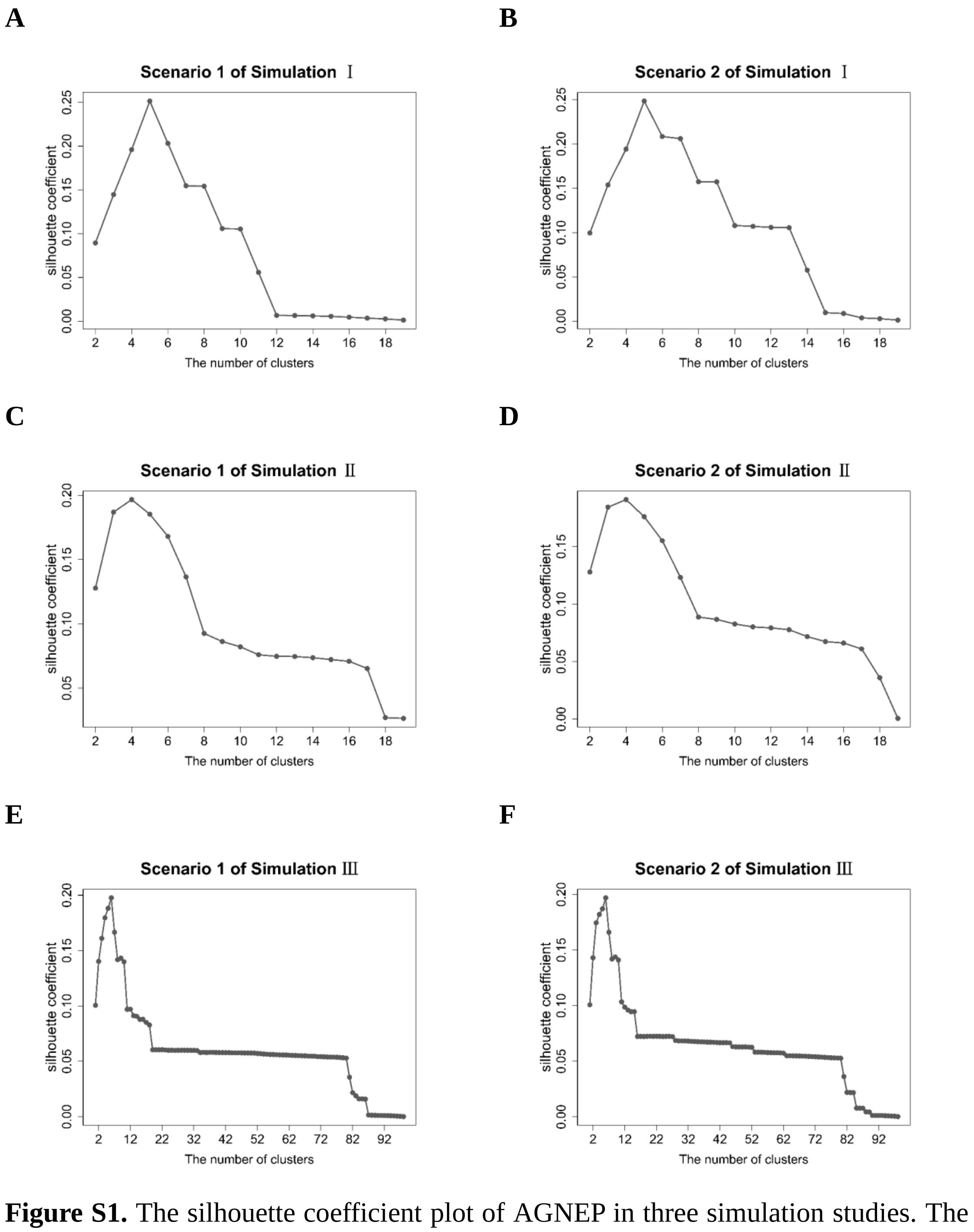


**Figure S1.** The silhouette coefficient plot of AGNEP in three simulation studies. The silhouette coefficient plots of simulation experiment I are presented in (**A**) and (**B**). The silhouette coefficient plots of simulation experiment II are presented in (**C**) and (**D**). The silhouette coefficient plots of simulation study III are presented in (**E**) and (**F**). Scenario 1 and 2 in the three simulation experiments indicate 10 QTNs and 50 QTNs are simulated, respectively.

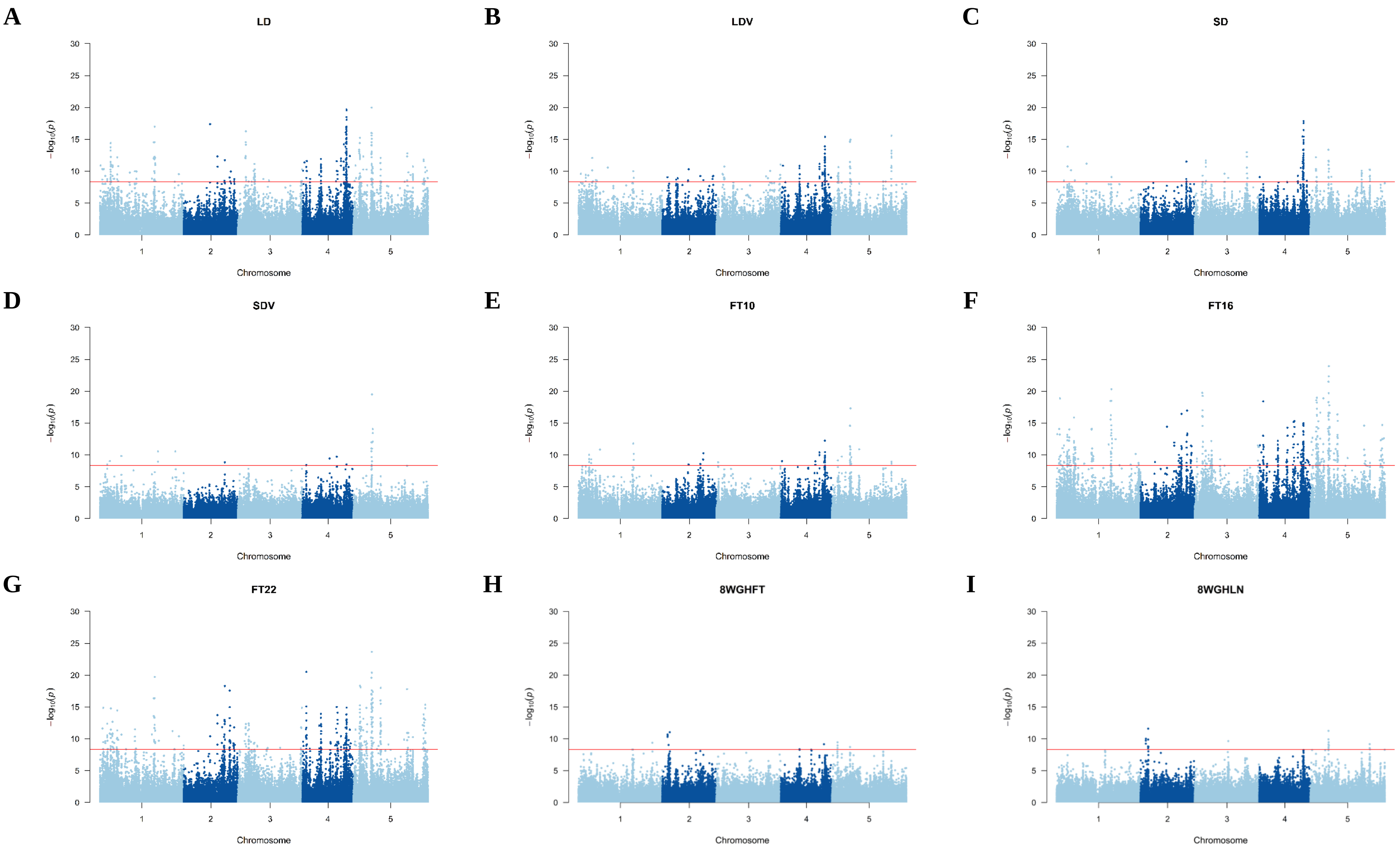

A
LD
B
LDV
C
SD
D
SDV
E
FT10
F
FT16
G
FT22
H
8WGHFT
I
8WGHLN
−log10(p)
Chromosome

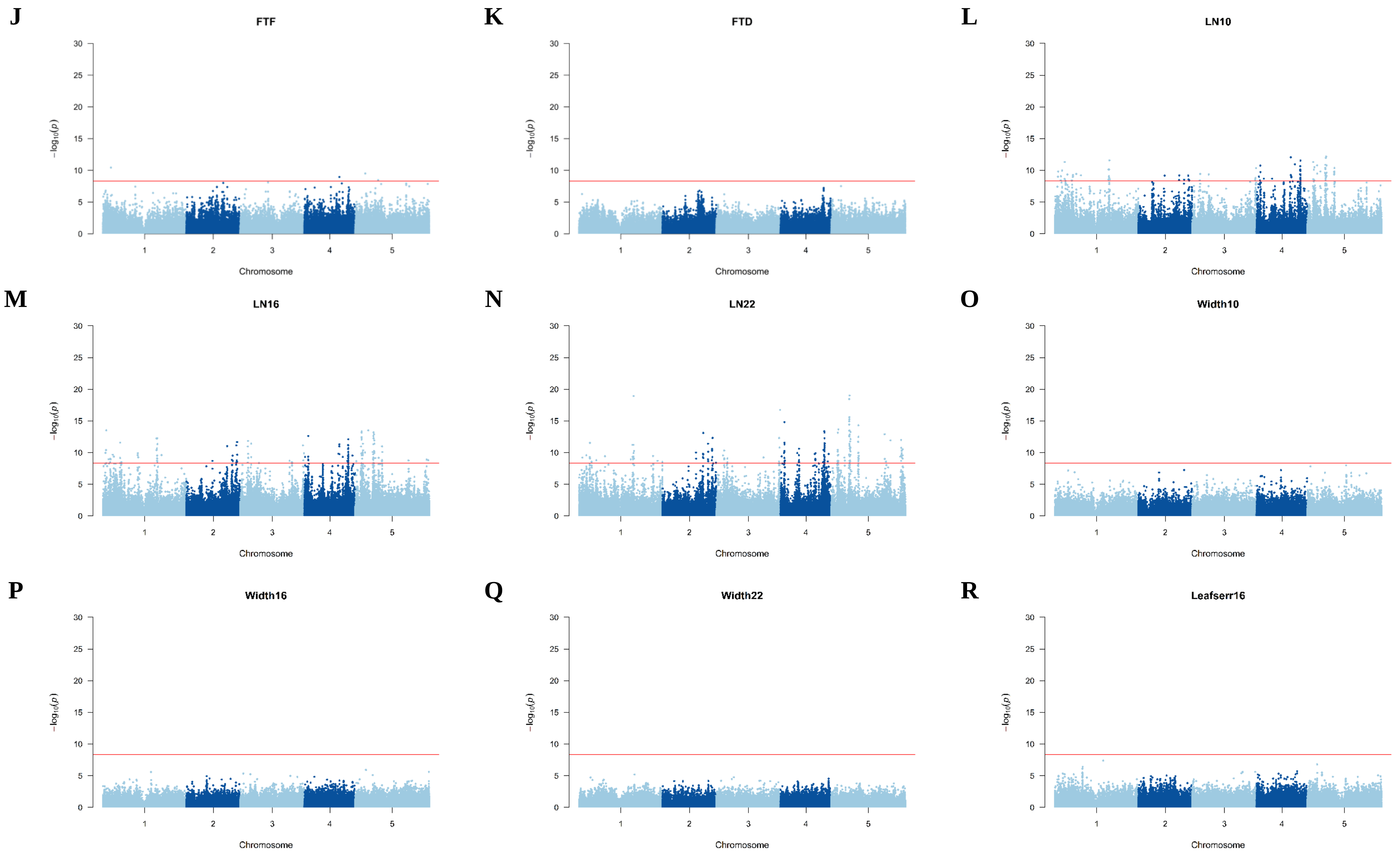
J
FTF
K
FTD
L
LN10
M
LN16
N
LN22
O
Width10
P
Width16
Q
Width22
R
Leafserr16
$-\log_{10}(p)$
0
5
10
15
20
25
30
1
2
3
4
5
Chromosome

**S**

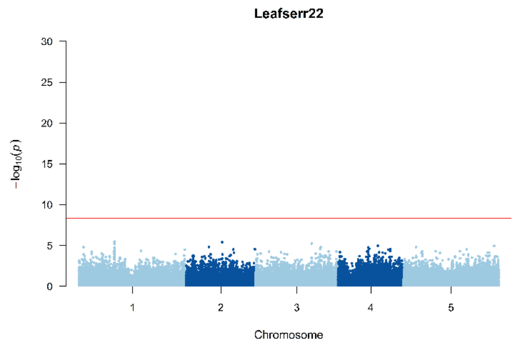


**Figure S2.** The Manhattan plot of the univariate analysis (ANOVA) for 19 traits in*Arabidopsis* dataset. The red line of each plot represents the Bonferroni corrected threshold of the test statistic, $-\log_{10}(0.001/206603) = 8.3151$.

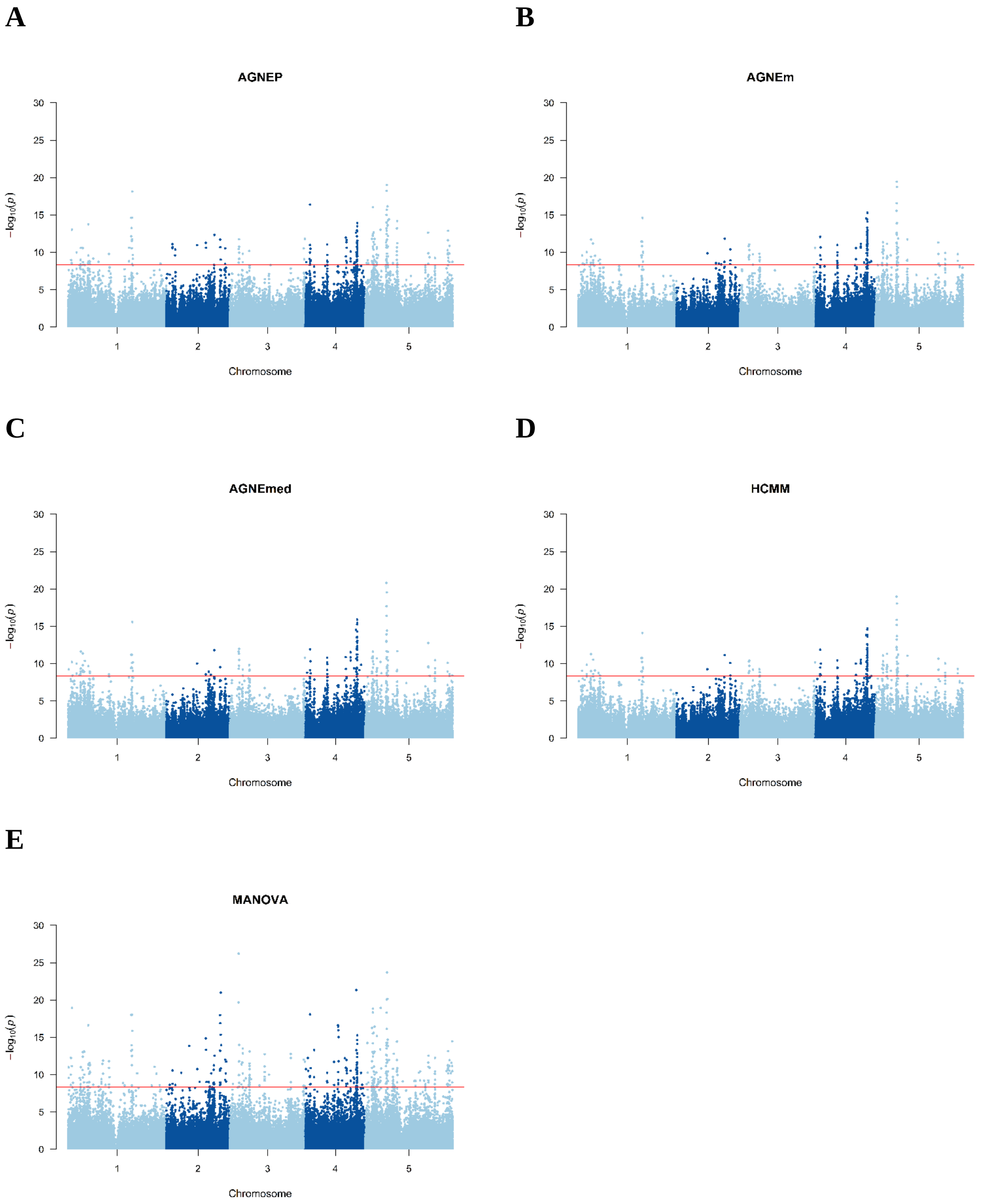


**Figure S3.** The Manhattan plot of six multivariate analysis methods (**A**) AGNEP (**B**) AGNEm (**C**) AGNEmed (**D**) HCMM (**E**) MANOVAin*Arabidopsis* dataset. The red line of each plot represents the Bonferroni corrected threshold of the test statistic, $-\log_{10}(0.001/206603) = 8.3151$.

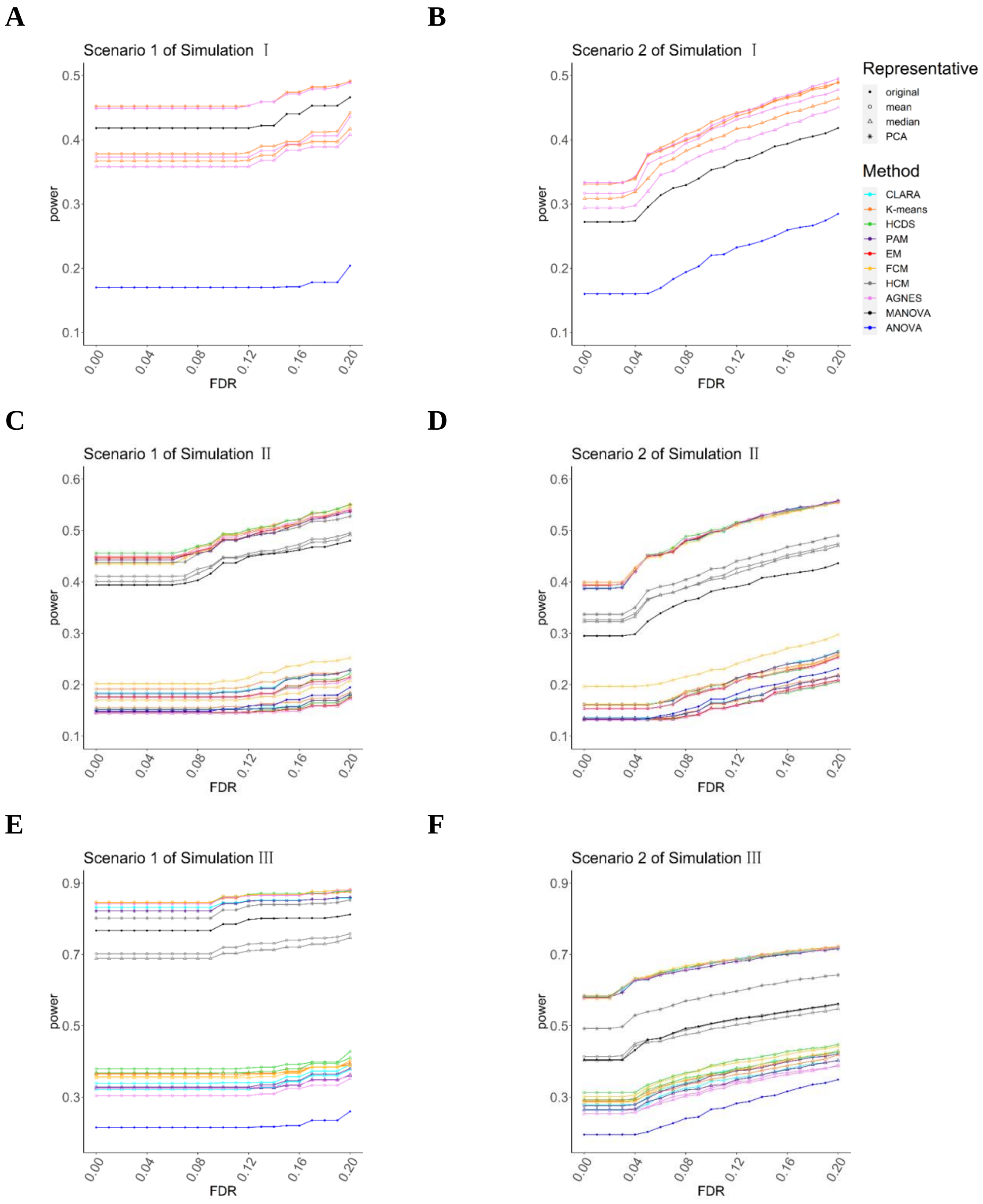


**Figure S4.** The power of different methods for three simulation experiments. In simulation I, the clustering results of AGNES, CLARA, HCDS, PAM, EM, FCM and HCM algorithm are same, thus these curves are overlapped anddisplayed as violet curves. The powers of simulation experiment I are presented in (**A**) and (**B**). The powers of simulation experiment II are presented in (**C**) and (**D**). The powers of simulation study III are presented in (**E**) and (**F**). Scenario 1 and 2 in the three simulation experiments indicate 10 and 50 QTNs are simulated, respectively.

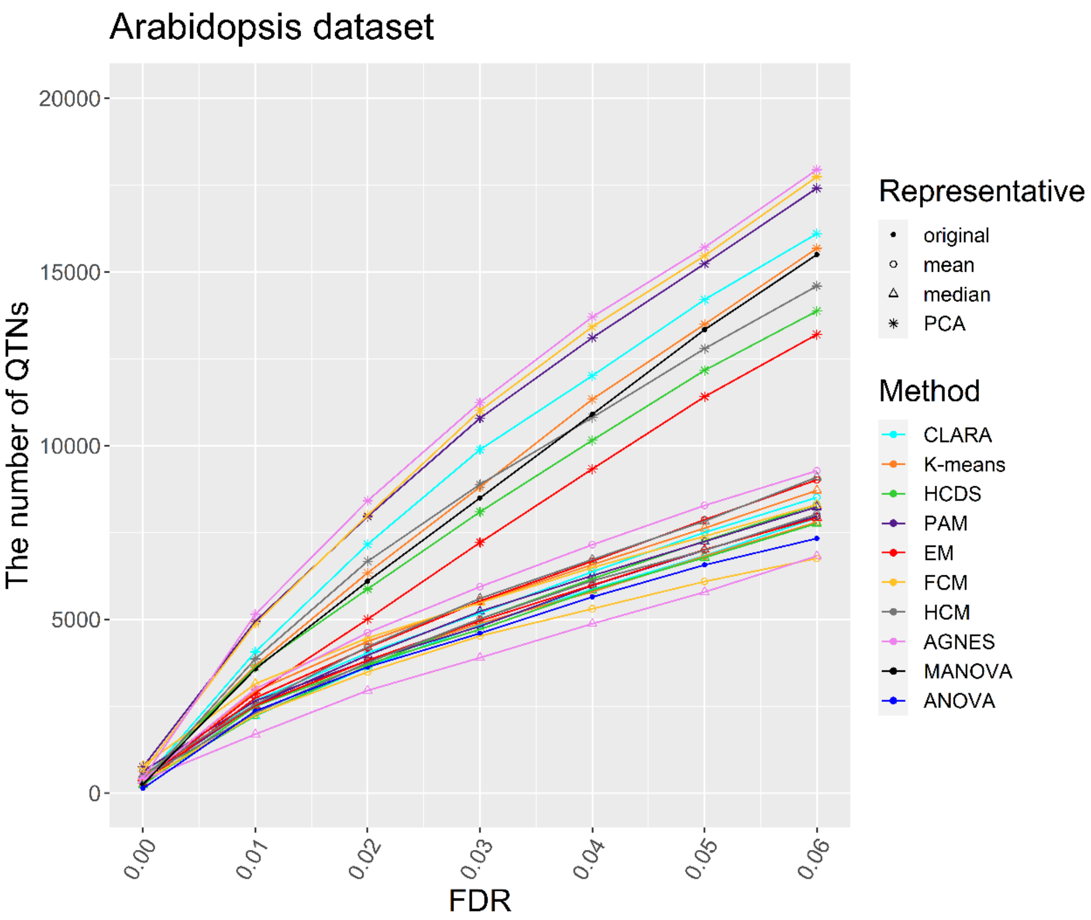


**FigureS5.** The number of QTNs under various FDR of different methods for *Arabidopsis* dataset.

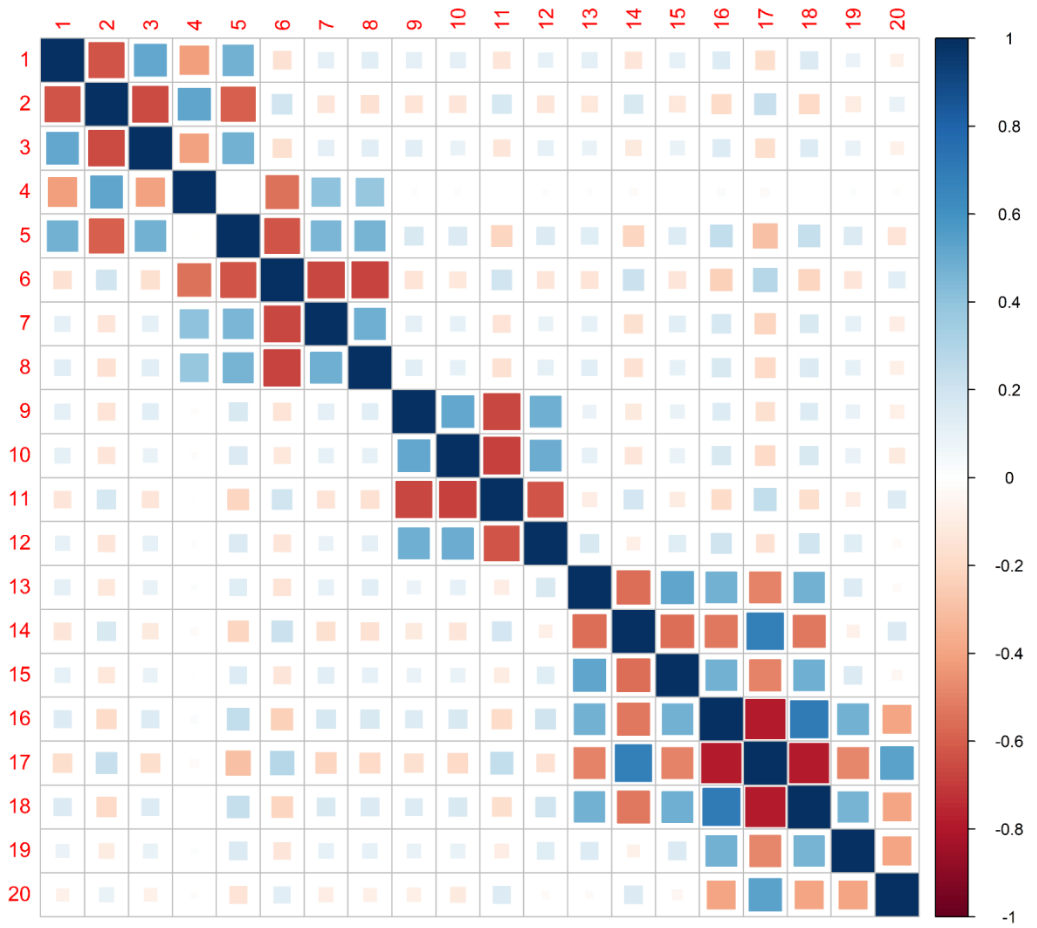


**Figure S6.** The genetic correlation for simulation experiment with mixed correlation under simulation II structure.

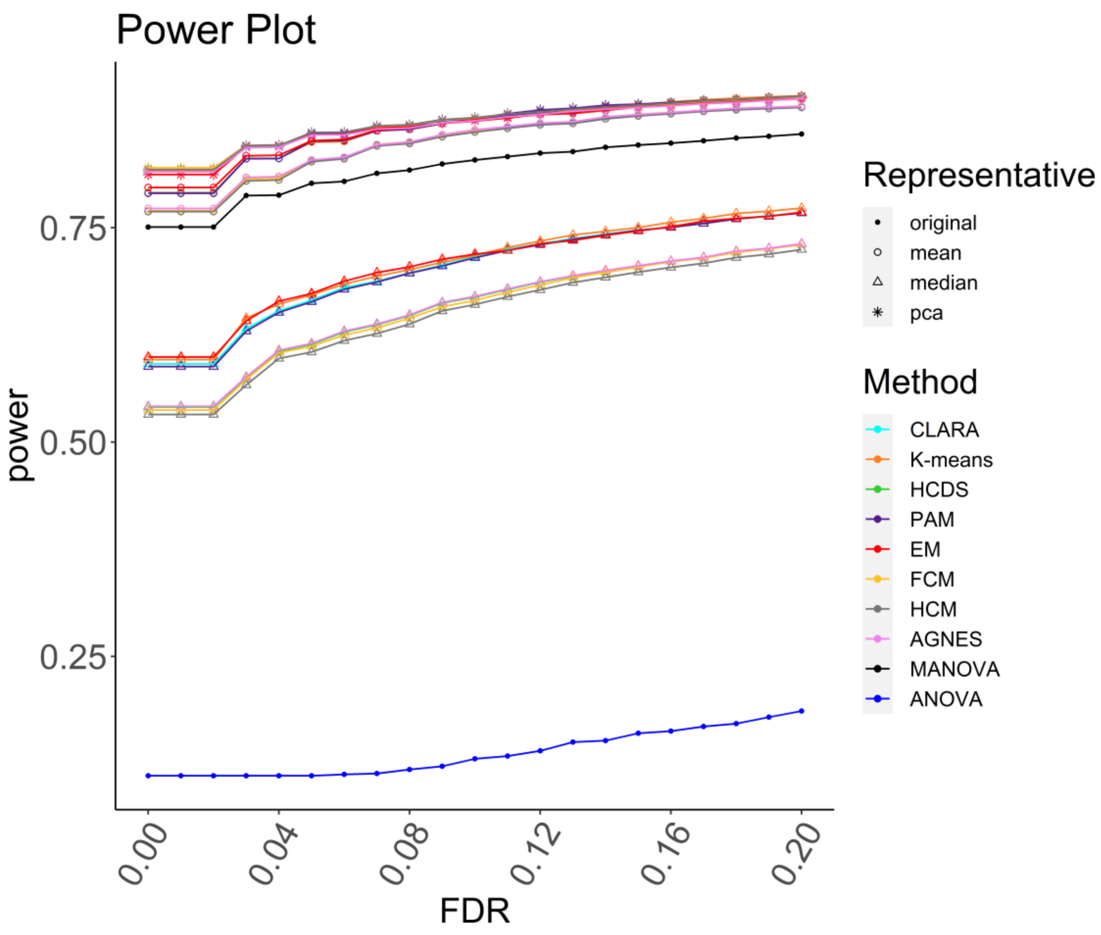


**Figure S7.** The power of different methods for simulation experiments with mixed correlation under simulation II structure.

# Supplementary Tables

**Table S1.** Average computational time (in minutes) of different methods for simulation experiments and *Arabidopsis* real datasets.

| Methods | | Simulation I | | Simulation II | | Simulation III | | *Arabidopsis* |
|---|---|---|---|---|---|---|---|---|
| | | **Scenario1** | **Scenario2** | **Scenario1** | **Scenario2** | **Scenario1** | **Scenario2** | **real datasets** |
| ANOVA | | 5.07 | 5.11 | 5.11 | 5.13 | 32.41 | 32.46 | 788.15 |
| MANOVA | | 0.83 | 0.84 | 0.82 | 0.84 | 12.05 | 12.29 | 110.72 |
| PCA | AGNES | 0.63 | 0.62 | 0.65 | 0.64 | 7.71 | 5.96 | 91.33 |
| | CLARA | 0.63 | 0.63 | 0.60 | 0.61 | 7.04 | 7.14 | 98.24 |
| | K-means | 0.63 | 0.65 | 0.65 | 0.64 | 11.25 | 11.24 | 103.14 |
| | HCDS | 0.64 | 0.64 | 0.61 | 0.61 | 7.79 | 5.94 | 112.46 |
| | PAM | 0.64 | 0.63 | 0.56 | 0.59 | 6.80 | 5.93 | 113.15 |
| | EM | 10.14 | 12.63 | 9.50 | 10.09 | — | — | 113.53 |
| | FCM | 0.63 | 0.61 | 0.59 | 0.60 | 2.62 | 2.64 | 105.23 |
| | HCM | 0.63 | 0.61 | 0.66 | 0.69 | 2.87 | 2.82 | 105.15 |
| Mean | AGNES | 0.39 | 0.40 | 0.44 | 0.44 | 4.82 | 4.41 | 113.49 |
| | CLARA | 0.38 | 0.39 | 0.39 | 0.41 | 5.51 | 5.54 | 106.78 |
| | K-means | 0.39 | 0.40 | 0.40 | 0.41 | 9.88 | 9.88 | 103.40 |
| | HCDS | 0.38 | 0.40 | 0.40 | 0.41 | 6.40 | 4.57 | 100.15 |
| | PAM | 0.38 | 0.39 | 0.37 | 0.41 | 5.41 | 4.56 | 103.81 |
| | EM | 9.88 | 12.40 | 9.30 | 9.89 | — | — | 93.86 |
| | FCM | 0.37 | 0.38 | 0.38 | 0.40 | 1.22 | 1.27 | 102.71 |
| | HCM | 0.38 | 0.37 | 0.74 | 0.80 | 2.52 | 2.48 | 105.05 |
| Median | AGNES | 0.40 | 0.41 | 0.46 | 0.45 | 4.86 | 4.43 | 95.64 |
| | CLARA | 0.39 | 0.41 | 0.40 | 0.42 | 5.53 | 5.57 | 104.04 |
| | K-means | 0.40 | 0.41 | 0.42 | 0.42 | 9.89 | 9.90 | 111.52 |
| | HCDS | 0.40 | 0.42 | 0.41 | 0.41 | 6.41 | 4.59 | 109.25 |
| | PAM | 0.39 | 0.40 | 0.37 | 0.41 | 5.43 | 4.59 | 106.55 |
| | EM | 9.90 | 12.41 | 9.31 | 9.90 | — | — | 101.78 |
| | FCM | 0.39 | 0.39 | 0.38 | 0.40 | 1.25 | 1.29 | 106.37 |
| | HCM | 0.39 | 0.38 | 0.81 | 0.85 | 2.75 | 2.67 | 106.96 |

Note. In the case of simulation III, EM clustering algorithm is not be considered because of the huge memory space requiredand long computing time.

**Table S2.** The number of significant SNPs and confirmed genes in *Arabidopsis* real datasets analysis under different methods.

| Methods | | SNP | Gene |
|---|---|---|---|
| ANOVA | | 133 | 159 |
| MANOVA | | 255 | 315 |
| PCA | AGNES | 433 | 453 |
| | CLARA | 340 | 378 |
| | K-means | 366 | 400 |
| | HCDS | 348 | 318 |
| | PAM | 754 | 724 |
| | EM | 348 | 381 |
| | FCM | 627 | 612 |
| | HCM | 276 | 321 |
| Mean | AGNES | 379 | 386 |
| | CLARA | 375 | 382 |
| | K-means | 471 | 477 |
| | HCDS | 316 | 318 |
| | PAM | 628 | 598 |
| | EM | 273 | 271 |
| | FCM | 339 | 340 |
| | HCM | 440 | 439 |
| Median | AGNES | 365 | 373 |
| | CLARA | 352 | 357 |
| | K-means | 451 | 451 |
| | HCDS | 236 | 235 |
| | PAM | 385 | 383 |
| | EM | 529 | 512 |
| | FCM | 787 | 742 |
| | HCM | 526 | 526 |
